# Incorporating local stakeholders’ voices and knowledge into conservation decisions: a case study on the Chinese Hwamei (*Garrulax canorus* Linnaeus, 1758) in Taijiang, Guizhou, China

**DOI:** 10.1186/s13002-022-00559-z

**Published:** 2022-10-14

**Authors:** Chuanyin Dai

**Affiliations:** 1 Key Laboratory of Ecology of Rare and Endangered Species and Environmental Protection (Guangxi Normal University), Ministry of Education, 1 Yanzhong Road, Guilin, 541006 China; 2 Guangxi Key Laboratory of Rare and Endangered Animal Ecology, Guangxi Normal University, Guilin, 541006 China

**Keywords:** Bird keeping, Community-based approach, Conservation policy, Cultural practice, Ethnoornithology, Local ecological knowledge, Stakeholder

## Abstract

**Background:**

The Chinese Hwamei (*Garrulax canorus* Linnaeus, 1758) is a widely distributed species and has long been kept as a pet, especially by the ethnic communities in Southwest China. According to conservation experts’ suggestions, it has been designated as a second-level national key protected species in February, 2021 to protect this bird, indicating that keeping it at home is no longer permitted in China. However, a key factor to ensure effectiveness and success of conservation initiatives is local stakeholders’ acceptance and support.

**Methods:**

Interviews and focus group discussions were used to document the policy outcomes and the views of 108 local bird-keepers in a county in Guizhou province.

**Results:**

Despite awareness about the illegality of the practice, the bird was still commonly caged both in rural and urban regions. To justify their unwillingness to stop keeping these birds, the interviewees presented many arguments, such as benefits for the community members’ health, cultural heritage and contributions to local livelihoods. Fewer than 30% of the bird-keepers believed that the practice of self-keeping has reduced the wild population. Most argued the decline was mainly generated by the harvesting and keepers with monetary interests. They suggested enforcement should target those people and bird markets, as well as the harvesting methods. They also recommended restricting the number of birds allowed to be kept by one keeper, establishing protected areas and a harvesting ban period. The study participants demonstrated considerable local ecological knowledge about approaches for managing the species’ use.

**Conclusions:**

Due to the benefits for the people and the bird’s large distribution, I argued that a conservation goal to lower the harvesting and keeping rates would be more appropriate than a strict ban on keeping them. Such a policy would be more feasible and culturally acceptable because it is built on keepers’ support and suggestions. It is necessary to monitor the effects of bird keeping on the wild population. Overall, this qualitative study demonstrated the advantage of factoring in local voices in conservation decisions.

## Introduction

Birds are one of the most hunted and traded animal groups worldwide since they are exploited in multiple ways in many human societies [1]. Particularly due to their attractive coloration, melodious songs and appealing behaviors, birds are kept in captivity as pets and ornaments all over the world, especially in Southeast and East Asia [2–4], and South America [5–9]. It has been estimated that the total number of bird species being kept in cages represent up to 37% of the world’s identified species [5, 10]. However, along with other factors including habitat fragmentation, degradation and loss; climate change; urbanization; pollution and natural disasters [10–12], keeping birds in captivity has led to population declines and threatened many targeted species [5]. Therefore, a wide range of measures, such as law enforcement to tackle illegal poaching and trade, setting up protected areas, restoring degraded ecosystems and controlling invasive species, have been adopted to combat these serious biodiversity concerns [13, 14]. However, to ensure the effectiveness and success of these conservation measures, the local public’s acceptance and support for those conservation initiatives need to be considered [15–19]. Acceptance and support among the key stakeholders are especially important [20, 21]. Obviously, if conservation management measure restricts the local people from using a natural resource they depend on for their livelihoods, it is more likely the conservation goals will not be achieved. This awareness has in recent years led to a growing number of research studies assessing local people’s attitudes toward conservation policies and programs [22–26] and developing community-based measures, by which local communities have net incentives toward supporting wildlife conservation [27]. For example, implementing community-based management has achieved a great ‘ecological success’ in Tanzania, resulting in significantly higher densities of resident wildlife observed in management area than the control site [28].

The Chinese Hwamei (*Garrulax canorus* Linnaeus, 1758) is a passerine of medium body size, widely distributed in South and Southwest China, North Laos and Vietnam [29], and an introduced population has established in Japan [30]. Isolated populations in the Hainan and Taiwan islands are regarded as separate species, respectively, according to recent molecular studies [31, 32]. This bird has been widely kept as pet due to its melodious song, elegant body and is sometimes being used for bird fights, but usually only the male is caged. Keeping Chinese Hwamei was common both in and outside of its natural distribution [33], as this bird has been recorded in markets out of its distribution, for example, in northern China [34] and Indonesia [33].

Keeping the Chinese Hwamei in captivity is popular in several ethnic mountain communities in Southwest China, such as in Guizhou and Yunnan Provinces, especially the Miao ethnic population, which has a long history and reputation of keeping wild birds as pets. In these regions, a pet Chinese Hwamei is claimed to be the best companion in a man’s daily life; for example, when he is at home alone, attends local open fairs, visits his relatives and friends, or even farms in the fields. At present, there is little literature investigating the scale of keeping and trading of Chinese Hwamei at local and regional levels in China, especially through household investigation. However, a few reports have found that numerous birds are traded and caged in both urban and rural areas, for example, in the city of Guiyang [2, 35, 36]. This city is the provincial capital of Guizhou province and is notable for the high numbers of wild birds being kept and traded; more than two hundred wild bird species have been recorded on sale in one of its public markets [36], and the Chinese Hwamei was one of the most coveted species with extremely high numbers [2, 35].

The large number of individuals on sale in the markets and being kept as pets has raised serious concerns among conservationists for the wild population, and has recently led to emergence of a conservation policy protecting Chinese Hwamei. At the beginning of 2021, the amended species list of the national key protected animals in China incorporated and conferred second-level protection status for this species [37]. According to the Law of the People's Republic of China on the Protection of Wildlife, it is prohibited for local people to capture and keep a second-level protected species in captivity without a permit (Article 21). Only competent research institutes with specific purposes, such as captive breeding and scientific study, can obtain permits from the relevant governmental agency (Article 25). Anyone who violates these regulations can face severe penalties (Article 45). For example, anyone who captures and keeps this bird can be subject to penalties including fixed-term imprisonment ranging from six months to several years, and additional large fines. Even before Chinese Hwamei’s elevation to conservation status, the media have frequently reported cases of people receiving penalties for capturing this bird, mostly in areas outside Guizhou province since it had already been categorized as a provincial key protected species in several provinces. In some cases, the courts judged the hunters guilty of prohibited activities as it listed in Appendix II of the Convention on International Trade in Endangered Species of Wild Fauna and Flora. However, such convictions are only proper if the bird has an exotic origin.

Similar to the findings in other regions [3, 4, 6, 8, 38, 39], the practice of bird keeping is a deeply rooted cultural activity in China. From an evolutionary viewpoint, human beings have developed a special emotional affiliation with companion animals through a history of co-survival and adaptive processes [40, 41]. This bio-cultural sediment has already evolved to be a central component of human life [41]. Accordingly, a previous investigation found that the Chinese Hwamei has cultural and social significance in the Miao ethnic community in Guiyang city [2]. Therefore, one pressing question needing to be addressed is whether such a conservation policy can win social support in these local populations. To this end, I carried out an investigation using ethnozoological approach in a county inhabited mostly by the ethnic Miao population in the southeast Guizhou province, where the bird may have a higher cultural significance since this bird-keeping practice has existed for a very long time.

As such, the goals of this study were: (1) to investigate the effectiveness of the current conservation policy by observing whether or not the local bird-keepers have stopped raising Chinese Hwamei; (2) to record local people’s attitudes toward the current conservation policy; (3) to document bird-keepers’ arguments and suggestions for conservation management; and (4) to formulate culturally compatible and feasible conservation activities by incorporating local people’s ecological knowledge and suggestions.

## Material and methods

### Study area

**Fig. 1:**
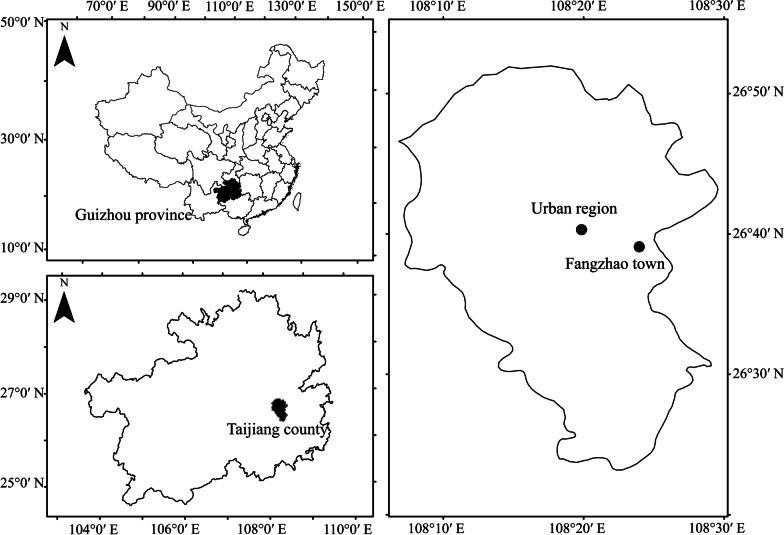
Map showing the location of the study area. The rural villages surveyed were within the district of Fangzhao town

Taijiang County is located in the southeast of Guizhou province, China (Fig. 1). It is a mountainous region with an area of approximately 1,108 km^2^ and an average altitude of 717.5 m above sea level. The climate is subtropical humid with an average annual precipitation of 1,108 mm and an average annual temperature of 16.5 °C. The forest coverage is up to 60%, and the vegetation primarily consists of large areas of conifer forests but also patches of evergreen broadleaved forests and shrubs. This county is famed for having the highest percentage of Miao ethnic population in China. The Miao community makes up 97% of the total population of approximately 0.13 million.

### Field work and data analysis

The survey was carried out from July 15 to August 24, 2021. A local male high school teacher in biology was hired as a field guide because he is from the Miao community and speaks the local language. The households in six rural villages (approximately 300 households) were investigated and the observed bird-keepers were invited to conduct interviews. However, most of the keepers declined to participate in these conversations, despite that the guide had explained our job, and the aim and nature of the interviews. This is understandable because keeping the birds had already become illegal in the survey period. Furthermore, the local government agency had carried out enforcement before our survey and thus the bird-keepers were afraid of law enforcement and further penalties. As a result, only three keepers accepted the interview invitation but hesitated to provide their personal information, such as age and education background and they initially all claimed that they did not know the Chinese Hwamei is a national protected species. Finally, a keeper recommended that we conduct interviews in the urban region of Taijiang County because the keepers meet in the forest near the urban region in the morning, suggesting that they may be more willing to discuss the topic in this outside setting. Following this suggestion, we carried out the following investigation in the urban region of the county. Due to unwillingness to share their personal information, the survey design was adapted to be a qualitative study. The results were based on verbal data from the interviewees, who used textual phrases and visual descriptions to state their opinions.

Initially, we also invited local people without pet birds to share their opinions on the question; however, only a very small number were willing to participate, giving the reason that they had no idea on the question as they did not raise this bird. Therefore, purposive sampling was used [42], whereby the observed bird-keepers were intentionally selected as they were likely to be most informative. The data were collected through in-depth personal interviews and focus group discussions, which are the two major instruments in social and cultural anthropology [43], and can be used in conservation social science [44, 45]. Semi-structured interviews were conducted through free conversation when a keeper accepted the invitation alone, while the focus group discussion methodology was adopted when two or more keepers were willing to share their views at the same time. Before the formal interviews or discussions, informed consent was obtained to record the conversations. To obtain useful information, we approached these bird-holders in a sincere and friendly manner and informed them of our occupations and the purpose of the investigation. All the interviewees were guaranteed that the personal information would be kept confidential if they provided.

Four open-ended questions were involved in the interviews. We firstly asked the interviewees whether they knew the status of Chinese Hwamei as a second-level national key protected species and the prohibition on caging. Then, we asked whether they support this policy and their reasons. We also asked about their perceptions of the population trends of the Chinese Hwamei in the surrounding and their reasons. At last, we asked those interviewees unwilling to stop keeping the birds, to suggest measures to reconcile the needs of bird-keepers and the protection of the Chinese Hwamei. In total, 108 bird-keepers and 5 non-keepers were involved in this study. Only one female, a non-bird-keeping high school student, was included in the study.

Grounded theory was used to analyze the above open-ended questions [43], from which the emerging themes and the relationships among themes were identified by means of open coding. This method identifies themes and patterns based on the text as they are observed during data analysis, rather than categorizing themes prior to coding data [43]. In short, descriptive codes were assigned to fragments of text according to normative statements, interesting facts and areas of disagreement, and the central idea of each response was categorized. Each category was determined twice to ensure the original data supported these categories. Codes may group hierarchically with a few top-level codes that represents the key themes.

## Results

### Bird-keeping practices

Caged Chinese Hwamei were observed in all six villages visited, with up to six individuals recorded in one house at most. We also observed empty cages in some households. Furthermore, the three interviewed keepers told us that most of the elderly in their villages raised this bird, while the high school student claimed that every household in her village had caged Chinese Hwamei and that her father raised them. However, we did not count the exact number of caged birds in these villages since it is hard to do.

**Fig. 2:**
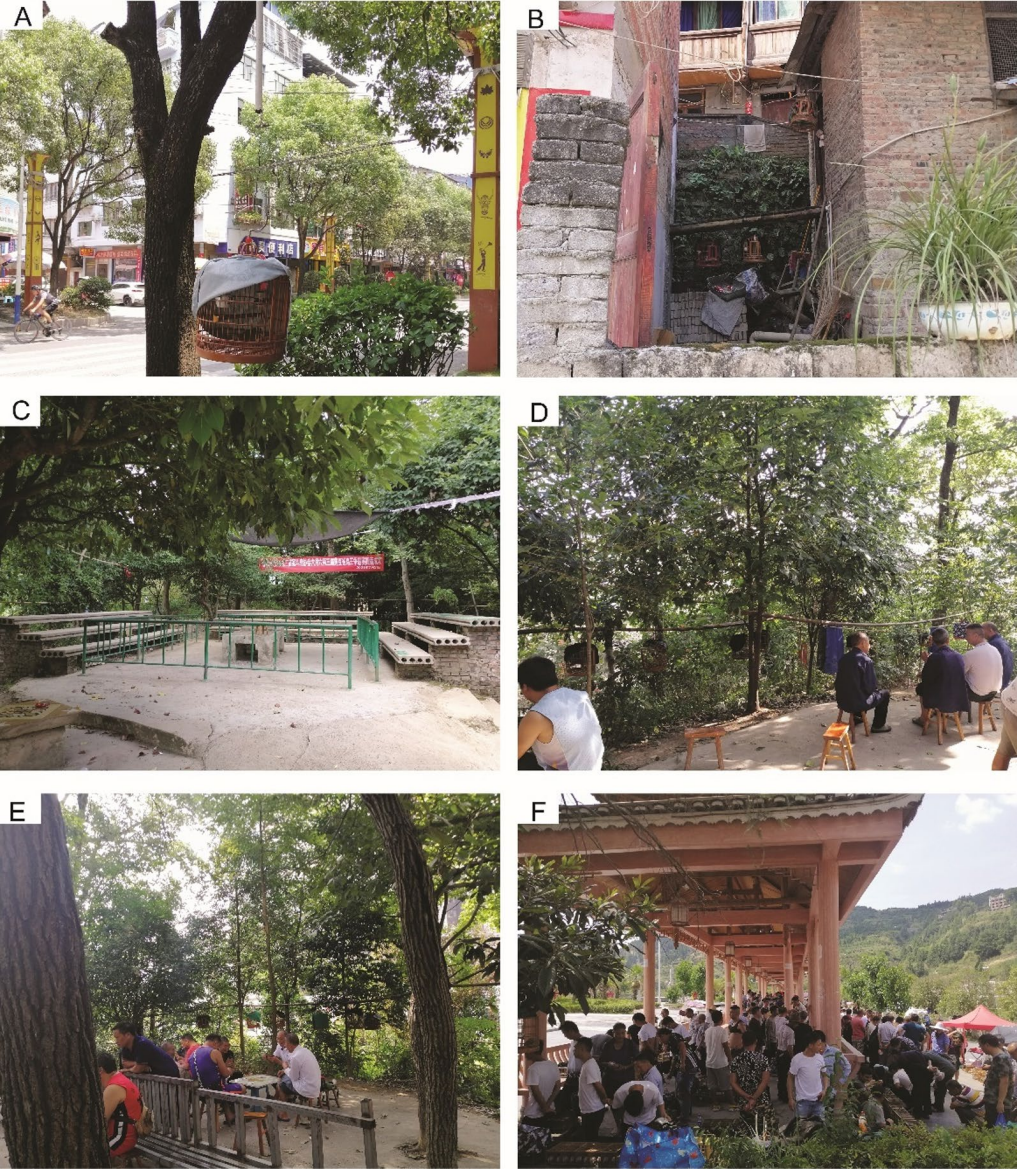
The still common practice of keeping the Chinese Hwamei and associated practices in the surveyed region: **A** A caged bird hanging in a tree in an urban street. **B** Four individuals kept in a residential house. **C** Gathering ground for bird fighting. **D** The keepers talking with each other at the morning gathering. **E** The bird-keepers playing poker at the gathering ground. **F** Many people at the open market held on Saturdays

In the urban areas, caged birds were more common, for example, in the tree-lined streets (Fig. 2A) and residents’ houses (Fig. 2B). Every day in the early morning, the keepers were observed to carry their pet birds and gather in the forest around their residences since the urban neighborhood was surrounded by small mountains. The gathering place was called ‘Niao Chang’ (鸟场) or ‘Dou Niao Chang’ (斗鸟场) in Chinese (Fig. 2C). At least 13 formal gathering places have been set up by the keepers. In the gathering places, the keepers hang their cages together. While the pet birds vocalized and sang, the owners chatted with each other or played poker (Fig. 2D and E). Bird fighting was also common. Most of the keepers returned home for lunch. In the urban areas, it was common to observe old men walking with their birds in the morning or at nightfall. Furthermore, an open-air market to trade the birds was regularly held every Saturday in an urban public square (Fig. 2F). Two parrotbills, *Paradoxornis webbianus* (Gould, 1852) and *P. alphonsianus* (Verreaux, 1870)*,* were also traded in high numbers.

### Awareness and arguments

All the bird-keepers already knew that the Chinese Hwamei is a national key protected species because the local governmental agency has informed them and required them to release their birds back to nature. However, most of the keepers considered this requirement as unacceptable and still raise their birds in cages. Only a few (*n* = 5) claimed that they would open the cages if there were strict enforcement, for example, a keeper said: ‘I will give up if they (the police) put me into prison.’

The keepers listed many justifications for this unwillingness to comply with the regulation. All the keepers emphasized that keeping the birds provides various benefits for the elderly, including the elderly themselves and younger keepers. It was said that the elderly always fall asleep when they stay alone or even watch TV at home, which was regarded as harmful for their health. In contrast, carrying their birds to participate the morning gathering can generate great benefits for their physical health due to the walking, mountain climbing and breathing fresh air. The elderly keepers also emphasized that their insistence on this practice has cured their illnesses, for example, two keepers said: ‘Keeping birds has cured my body pains due to rheumatism and arthritis.’ Without a pet bird, they would never walk into the forest as they thought the contact with forest and other birds is necessary and good for their pet birds. It is the needs of the bird that pushed them to outdoor activities. Furthermore, talking with the other keepers in the gathering ground was considered very important for maintaining their mental health as they found it difficult to communicate with younger people. Additionally, they felt very happy to watch their pet birds or listen the voices of the birds. Some of the respondents (*n* = 5) expressed the view that they need pet birds to pass the time as they have nothing else to do. In addition, it is worth mentioning that except for the female high school student, the other four non-keepers also thought that the elderly benefited from keeping birds, and that conservation should also consider their needs.

Most bird-keepers (*n* = 89, 82.41%) also argued that keeping the birds in cages is indeed a form of protection, which can benefit the birds in many ways. They explained the kept birds are not merely considered as pets. They actually love their birds more than themselves; for example, in the past during times of hardship, the birds were fed on chicken eggs, even when their children and they themselves could not eat eggs. Nowadays, they buy a variety of nutritional foods to feed their birds, such as beef, wild insects and the specialized composite feed. They sometimes collect wild insects themselves for feed. In order to highlight their love for their birds, one frequently cited statement is that a keeper will instinctively lift his bird to avoid damage when he slips over, even if this may cause serious injury to himself. Some keepers (*n* = 5) claimed that the life span of the pet bird is approximately 7–8 years due to their care, which is much longer than that of wild birds which is about 3–4 years. They also argued that keeping birds in captivity can protect them from natural predators, for example, hawks. Some bird-keepers (*n* = 7) declared they spent almost all their time and energy on raising and caring for their bird, and did nothing else, and others (*n* = 3) stated they always provided food for the wild birds in the gathering grounds or other forest areas.

Culture and cultural heritage (*n* = 78, 72.2%) also emerged as an important driver for the disagreement on the current conservation policy. Since their childhood, the bird-keepers had observed the caged Chinese Hwamei kept by their fathers and grandfathers. They claimed this practice has a history of at least 200–300 years. Furthermore, pet bird singing competitions and fighting, along with cow fighting, dog fighting and cock fighting, were always the highlights of the programs in the celebrations and ceremonies in their ethnic group. Cultural beliefs also promote the popularity of the bird-keeping activity. It is said that keeping the Chinese Hwamei can make the family thrive, or that the children will not perform their filial duty if an elderly man does not keep birds.

The keepers stated that keeping pet birds also has benefits for the younger keepers (*n* = 54, 50%), for example, fostering positive personal habits, such as getting up early, and avoiding gambling and drinking. One young man (35 years old) told it is the bird’s voice which makes him get up early because he feels the bird is telling him it is the time to carry HE (it) to the forest. Another young man (31 years old) claimed that he would be asked or invited to drink or gamble by his friends or business partners if he did not have birds to care for. Taking care of the birds is a perfect excuse to decline those invitations but still maintain these relationships. Additionally, they can also make friends with younger and elder keepers and build wide social contacts.

Finally, another frequently cited reason for the practice of keeping birds is the substantial contribution it makes to local livelihoods and economic growth (*n* = 44, 40.7%). For example, local people in poverty can gather and sell the wild edible insects and spiders, which are used by the keepers as live feed for the birds. There are also many people who sustain their livelihoods by selling hand-made bird cages, and the mixed feed they require.

Disagreements with the conservation policy were expressed based on other arguments with less mention. For example, some keepers claimed that the practice has no adverse effect on wild populations (*n* = 15, 13.8%) and thus should not be prohibited, whereas other keepers argued that it is impossible to prevent approximately 20,000 keepers from raising their birds in this county as they will fight for their rights (*n* = 2, 1.8%). One keeper responded that the bird-keeping activity can help control agricultural pest insects. As they buy live insects to feed their birds, the local people in poverty capture a large number of pest insects each year. Several keepers questioned whether the zoos could raise national protected species for viewing by the public (*n* = 5, 4.6%), and thus it was illogical to prohibit their practice of keeping birds since they can provide better living conditions than for the birds in the wild.

### Population trends

Only 32 bird-keepers regarded that the Chinese Hwamei population is or may be in decline around their residences and regarded the capture and keeping as the major reason for its decline. However, most of them (*n* = 25, 78.1%) attributed the decline to the harvesters and bird-keepers with monetary motivations. Other factors were also suggested as drivers for the population decline. For example, two interviewees believed that the increase of predators was the predominant reason as they had observed predators such as hawks and snakes eating eggs or nestlings. They claimed that the predators have increased remarkably due to the protection of the forest and environment. On the other hand, another bird-keeper believed the reason is the decrease of suitable habit. He observed that the Chinese Hwamei occurs in shrubs and woodland, which have sharply decreased in area due to natural community succession caused by the forest protection without human disturbance.

Most bird-keepers (*n* = 71, 65.7%) believed that the wild population is stable, and that bird-keeping activity has no or at most very little impact on the population structure and dynamics. They argued that the bird is only used as a pet and never for food. Unnatural deaths resulting from bird-keeping practices are almost impossible because the bird-keepers have sufficient expertise to ensure the comfortable living environment for birds in captivity. Once they do not want to keep it, the bird will be released back to nature. They claimed that the caged life has little adverse effects on the bird’s reproductive ability as the released birds have been observed to produce new generations around the gathering grounds. This was confirmed by observations of fledglings and adults feeding on the food provided by bird-keepers in the gathering grounds by the author of this study. Bird-keepers also argued that all the wild birds will die due to various circumstances, such as being predated, short life spans and food shortages, and these losses will be supplemented by newborns. Capturing wild birds for keeping in captivity could be regarded as a regulating factor to mediate competition among wild individuals. Furthermore, they claimed that a favorite pet bird can be kept several years (for example, 3–4 years), which provides sufficient time for the recovery of the wild population as they have found that the bird can breed in at least two to three nests in a breeding season with three to four eggs per nest. One bird-keeper argued that the caged birds were from domestically breeding populations, and thus do not affect the wild population at all.

Interestingly, a few bird-keepers suggested that the wild population is increasing in their surrounding area (*n* = 5, 4.6%). They argued that most of the captive birds were captured in other regions, rather than the local area, while the practice of releasing birds back to the wild added to the local population size.

### Suggestions

**Fig. 3:**
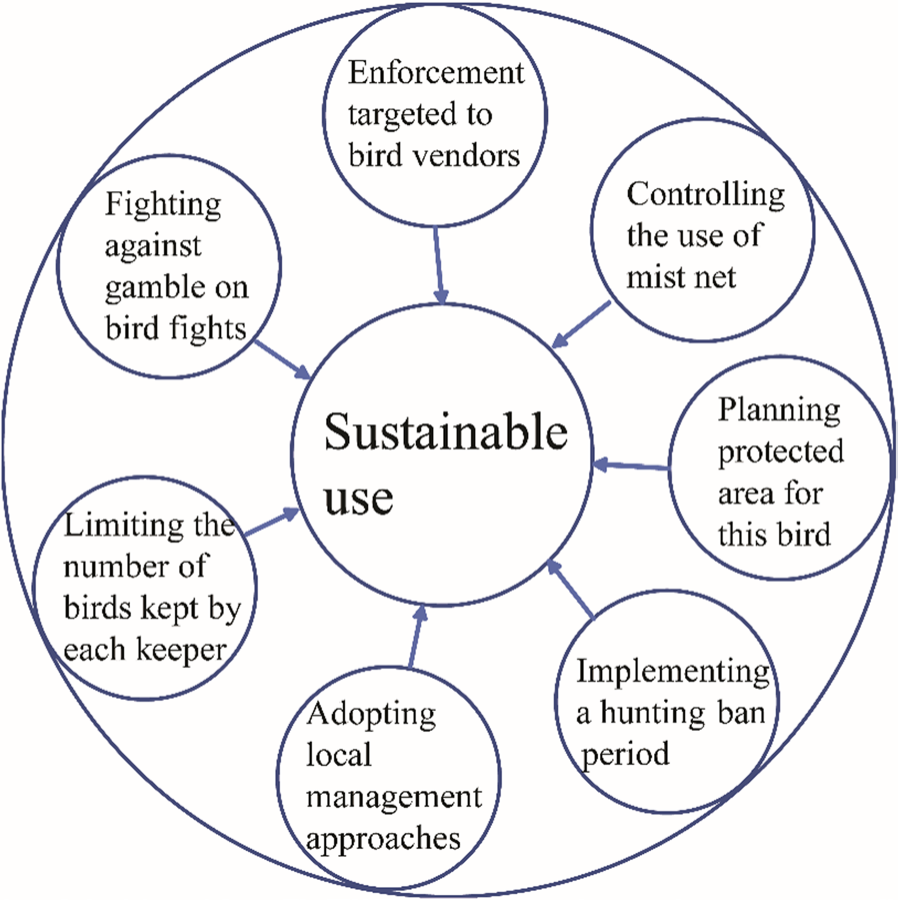
Suggestions provided by the local keepers to balance the need for the conservation of the Chinese Hwamei and bird-keeping practice

Several suggestions were made by the bird-keepers to balance the need for the conservation of the Chinese Hwamei and their bird-keeping practices (Fig. 3). They recommended that the number of birds kept by a keeper should be strictly controlled, for example, by allowing only one or two individuals to be kept. They acknowledged that a lot of bird-keepers raise too many birds, some up to ten individuals. In such cases, the death of pet birds could emerge because the bird-keeper has limited time and energy to take care of the birds. Therefore, taking too many birds from the wild population could affect the species’ persistence and regeneration.

They also suggested that enforcement should target the people who capture and keep birds for economic purposes because people harvesting birds for sale do not have any love for the birds but are only motivated by money and their activities could lead to high death rates during the process of capture and transportation. Accordingly, they argued that the market for the trade should be monitored and that people involved in the trade should face harsh penalties. Along the same lines, gambling on bird fighting should be prohibited, and those bird-keepers must also face harsh penalties. For those bird-keepers, the birds are only considered tools to earn money. One interviewee noted that he had observed an owner kill his bird immediately after the bird lost a match because the defeat caused him to lose money.

The bird-keepers also mentioned that the supply of mist nets must be restricted. The efficiency of capturing birds using nets has enticed many people to begin this practice as it can generate considerable income. They explained that hunters using nets hardly ever release the birds being captured, which not only causes damage to the Chinese Hwamei, but also other nontarget birds. Instead of mist nets, harvesters could use traditional tools such as foot snares and cages, which capture at most one bird at a time. If the wild population is in decline, few birds will be captured using such traditional tools. This can decrease the number of harvesters aiming to obtain high monetary returns because of the lower harvesting success.

Bird-keepers suggested setting up protected areas to protect the birds. Capture would not be allowed in these areas and violations must be taken serious penalties. Those areas could be managed as source stock for maintaining the wild population. Some bird-keepers proposed a hunting ban period to allow the wild bird population and captive population to rebalance. They claimed that a pet bird can be kept for several years and that it is not necessary to harvest wild birds every year. It would be effective to ban the harvesting activity for a regular period, in which time the wild population could generate offspring to offset the loss if any, due to bird keeping.

The keepers emphasized the continued abundance of Chinese Hwamei in their surrounding area despite of a long history of being kept is due to their own culture and approaches to managing the resource. For example, pet birds are never used as food. It is said that if a keeper eats pet birds, he or his offspring will someday go to prison. Rather, a pet bird should be released back to nature if a keeper stops raising it. Before release, the bird is always marked, for example, by trimming a little claw. If this bird is captured once again, the harvesters will check the mark and free it immediately. Most importantly, the birds around their houses are only allowed to be harvested by themselves or members of their communities. Strangers were prohibited to harvest these birds, because they were most likely the hunters with economic motives. They also only capture and keep male birds, whereas females are immediately released. In their view, this is the ideal approach to reconcile the conservation of the Chinese Hwamei and their bird-keeping practices.

## Discussion

### Current policy performance

The effectiveness of a conservation policy should be monitored to ensure the fulfilment of the planned or expected goals [46]. Our study showed that the current policy designed for the protection of the Chinese Hwamei has made almost no progress toward its goal of eliminating bird-keeping practices in an ethnic region. It should be mentioned that only a small number of residents were present in the rural villages during the time of the survey because many families sustain their livings by providing migrant labor in other areas. However, most of those present were the elderly, who are exactly the most likely group to raise the Chinese Hwamei in their homes. On the other hand, a large number of bird-keepers can be encountered in the urban region, and it is common to observe them gathering in the surrounding forests with their caged birds every morning, whereas an open market for trading wild birds is held weekly. This was quite unexpected since the system of law enforcement in conservation has been praised as efficient and effective in China [47]. The local governmental agencies have the legal power and right to administer penalties, and these responsibilities are inspected and supervised by the upper and top-level environmental agencies [47].

However, this failure should be understood within a complex social–cultural context, rather than simply attributed to insufficient enforcement. According to the bird-keepers, they have close emotional and physical affiliations with the Chinese Hwamei since it can bring them various benefits, especially for their health. Within this context, many studies have already highlighted the physical [48, 49], psychological [50, 51] and psychosocial [52, 53] benefits for the owners of companion animals [41], including pet birds [54]. At the same time, the pet birds can also benefit from, for example, better shelter and food provided by the keepers. They also believed that the bird-keeping practice contributes to the well-being of local low-income people, the economy, agricultural production and environmental protection. For example, the use of live insects as feed for the pet bird can make substantial contributions to the livelihoods of the low-income people who gather insects, as well as control pest insects, which leads to less pesticide use. In this regard, the contribution to the low-income insect gatherers has been recorded in a recent investigation in Guiyang city in which the selling of the Oriental mole cricket as live feed was the most important family income source for 82.4% of the interviewed gatherers [55]. Most importantly, most bird-keepers did not believe that their practices can cause any adverse effect on the wild population. Their logic is simple but impressive because the Chinese Hwamei is still common in their surroundings despite of a long history of keeping the birds in captivity. The ban on the use of the bird obviously did not take these underlying social and cultural drivers into consideration, resulting in undesirable outcomes. Since it is claimed there are approximately 20,000 bird-keepers in the county, strict enforcement of the restrictions would be very onerous. Serious conflicts have arisen when police tried to confiscate birds or required the keepers to release their birds as claimed by the interviewees. These situations are predictable as conservationists have already highlighted several serious limitations of the ‘top-down’ enforcement approach, such as imposing unjustified restrictions on the use of wildlife resources, infringing rights, increasing the costs of living and undermining the benefits to local people from wildlife conservation [27]. There are also plenty of studies highlighted that considering the social dimension and community acceptance is the key ensuring lasting success of a conservation management strategy [26, 38, 40, 47, 56, 57].

### Conservation suggestions

Identifying declines and the drivers in biodiversity to prevent species extinction is the predominant goal for conservation [58]. Its target is only to enhance the welfare of non-human biodiversity. However, recent developments have refreshed its intellectual and academic framework, and the core principle of biological or environmental conservation should now be flagged as conservation for people rather than from people [59]. This principle aims to find and promote strategies that can jointly maximize the protection of biodiversity and the improvement of human well-being [59]. Maintaining the wide range of contributions nature makes to people’s well-being has become a key goal for protecting nature [60]. Since this goal has incorporated local livelihood, economy and development into conservation, it can win wide support from the public, especially the local people.

The Chinese Hwamei has a very large distribution in East Asia and is currently assessed as Least Concern on the IUCN Red List of Threatened Species [29]. On the other hand, according to the bird-keepers, bird-keeping practices can generate well-being among people in many ways, especially among the elderly, and also some non-keepers. These benefits have also been reported in other studies [2, 55], indicating that these well-being benefits are not limited to a particular region. Given that one of main motivations for conservation is to safeguard well-being for human, it is better to consider a conservation strategy targeted to sustainable use rather than simple bans on use which relied heavily on environmental enforcement. The still common bird-keeping practice in the study region indicates that the ban on bird keeping is not effective because it has received extremely limited community support.

It has been suggested that dialogue and knowledge exchange among stakeholders is a necessary and important link for finding effective solutions and policy in environmental resources management [61–64]. In particular, the inclusion of various delineated human perspectives has proven useful and effective in many biological resource management and conservation areas [65–67]. These perspectives are derived from the accumulation of personal experiences in a dynamic environment, tested by a long process of trial and error [68] and are thus provide dynamic insights, skills and responsibilities for sustainable use and conservation of natural resources [47]. Therefore, factoring in the voices of local people and their cultural practices into conservation decisions is very important since it is a significant determinant of the outcomes of biodiversity conservation [47]. As such, current conservation goals could be adapted to lower the harvesting and bird-keeping rates which can be promoted with increasing public and social supports.

Although all the bird-keepers may harvest and keep Chinese Hwamei, it seems that they are a heterogeneous group with different motivations. Such differences among the harvesters and bird-keepers have also been reported in several other studies and require conservation efforts that acknowledge this distinction [69, 70]. The bird-keepers have proposed several measures to lower the harvesting and captivity rates of the Chinese Hwamei. For example, they argued that the people who harvest birds aiming to sell them and people who keep birds for gamble fighting should be blamed for the decline in wild population. The bird-keepers argued that enforcement should target those people. Efforts also should be made in monitoring the market for the trade of birds and targeting harvesters’ use of mist nets and multi-room cages. Some bird-keepers even suggested that the mist net should be controlled in production. For the bird-keepers without economic motives, they suggested that the number of birds being kept could be strictly controlled, allowing, for example, no more than two individuals for each keeper. The hunting ban areas and time periods were also recommended, since a pet bird can be raised for several years. Furthermore, empowering and engaging local communities in conservation could further help achieve this conservation goal [27, 71]. For example, the custom that prohibits strangers and outside harvesters from capturing the birds in and around the bird-keepers’ community and applying a mark when releasing pet birds back to nature could be useful for the sustainability of the wild population. All these suggestions could be considered by the relevant government agencies. Since these measures were proposed by the local stakeholders, it builds on the widest social supports and can quickly achieve the goal of lowering the bird harvesting and keeping rates.

Additionally, it is worth noting that the literature has presented captive breeding as a successful approach to deal with bird keeping [72]. However, it has been completely prohibited in China due to the suspected linkage between the emergence of coronavirus disease 2019 (COVID-19) and wild or farmed animals [73]. Yet, this approach deserves to be encouraged.

It should be mentioned that the trade in caged birds and keeping practices have been identified as a predominant driver for the scarcity of the Chinese Hwamei in Vietnam [74, 75]. However, no similar study thus far has been carried out in China. It is necessary to carry out scientific investigation focusing on the effects of bird-keeping practice on the wild population, such as population density and behavior change under different harvesting pressures. For example, flight initiation distance can be used as an indicator of hunting pressure [76]. With these indicators, enforcement could be carried out in a more efficient way by, for example, targeting regions displaying a high level of harvesting pressure. Furthermore, population genetic structure should also be monitored since the harvesters only keep males that are considered beautiful and good at singing or fighting. Obviously, whether such a selective harvesting can have genetic effects on the wild population is a paramount concern for conservation [77–79]. In addition, the locals’ views about the increase in predators and the decrease of suitable habitat for this bird merit for further scientific investigation. It is true that the shrubs and woodlands have been declining due to forest protection and lack of human activities (Personal observations). The high level of predation pressure could be related to the decrease in shelter for breeding, and if necessary, local keepers could be encouraged to engage in creating and enlarging the suitable habitat for this bird.

## Conclusions

Conservation policy always relied on the suggestions from conservationists and experts, as the case in the Chinese Hwamei in China. However, this ethnobiological study showed that the current policy aiming to prohibit the harvesting and keeping of Chinese Hwamei has had little influence in ethnic communities in Guizhou province, China. The bird-keeping practice is still common in both urban and rural areas because the local bird-keepers argued that this pet bird has a significant role in their daily life, and that the birds can also obtain benefits from being kept in captivity. They suggested that conservation measures should target people who harvest and keep birds for economic motives, and proposed various measures to control and lower the harvesting pressure on the species. Since keeping birds contributes to the well-being of bird-keepers and other local people, a conservation goal to lower the levels being harvested and kept would be more appropriate than a strict ban on these activities, especially in light of the large population distribution and size of the Chinese Hwamei. Furthermore, studies should be urgently conducted to investigate the effects of bird-keeping on the wild population, specifically on population density, behavior and genetics. In short, this study highlighted the role and potential contributions of local stakeholders in the sustainable management of natural resources [80] and the advantages of taking their voices and knowledge into consideration in conservation initiatives [47].

## Data Availability

The datasets used and/or analyzed during the current study are available from the corresponding author on reasonable request.
