# Correction: Incorporating local stakeholders’ voices and knowledge into conservation decisions: a case study on the Chinese Hwamei (Garrulax canorus Linnaeus, 1758) in Taijiang, Guizhou, China

**DOI:** 10.1186/s13002-022-00561-5

**Published:** 2022-11-04

**Authors:** Chuanyin Dai

**Affiliations:** 1 Key Laboratory of Ecology of Rare and Endangered Species and Environmental Protection (Guangxi Normal University), Ministry of Education, 1 Yanzhong Road, Guilin, 541006 China; 2 Guangxi Key Laboratory of Rare and Endangered Animal Ecology, Guangxi Normal University, Guilin, 541006 China

## Correction to: Journal of Ethnobiology and Ethnomedicine (2022) 18:63 10.1186/s13002-022-00559-z

Following publication of the original article [1], the author reported that an incorrect version of Fig. 1 had been provided, in which South China Sea islands had not been presented in the map.

The published article has now been updated with the correct version of the figure, and the corrected figure may be found in this erratum.

The author thanks you for reading this correction and apologizes for any inconvenience caused.

**Fig. 1:**
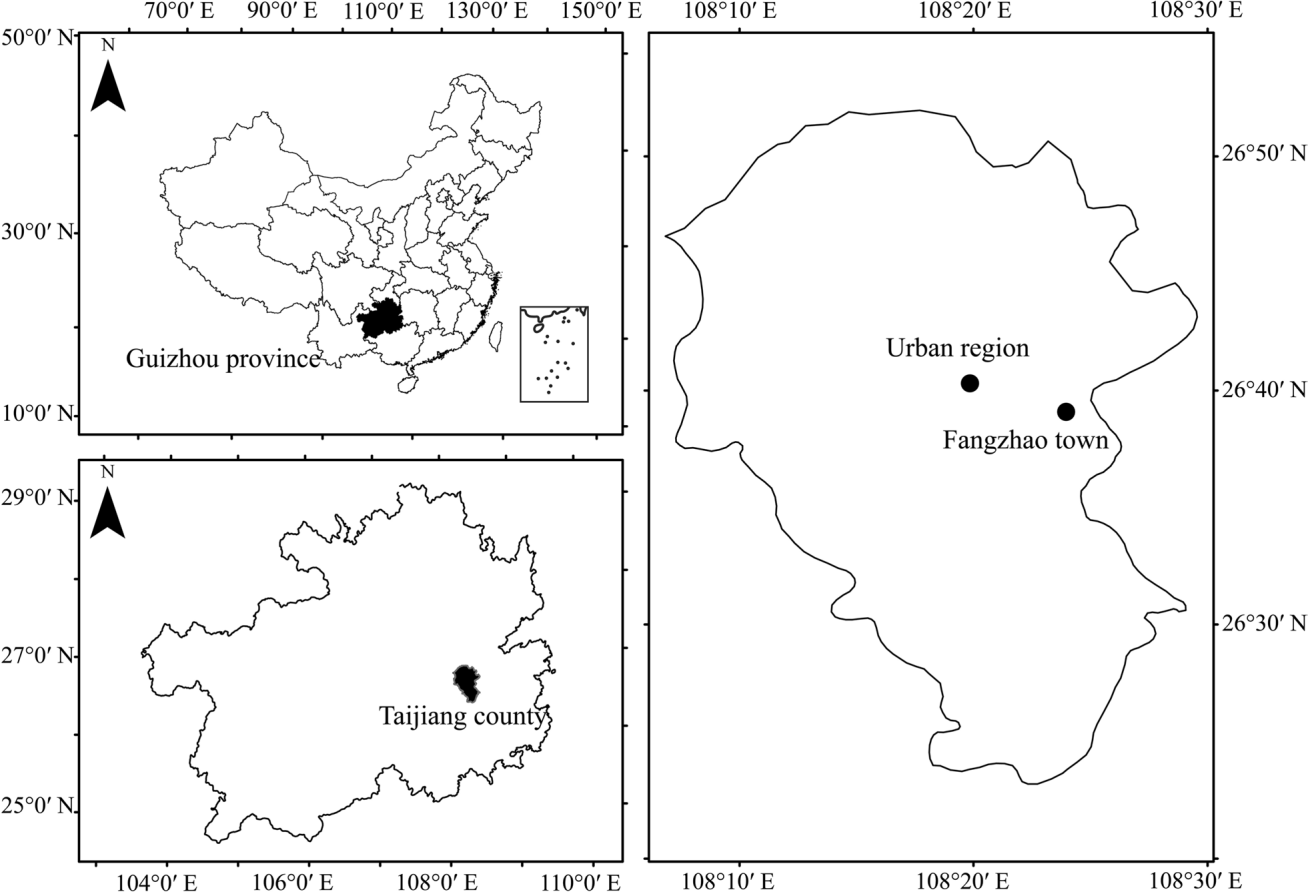
Map showing the location of the study area. The rural villages surveyed were within the district of Fangzhao town
